# Dilated Gallbladder Causing Right Iliac Fossa Pain in a Patient with Advanced Pancreatic Cancer: A Diagnostic and Symptom Management Challenge at End of Life

**DOI:** 10.1177/26892820261424786

**Published:** 2026-03-10

**Authors:** Sumayyah Omar Bintalib

**Affiliations:** Division of Supportive and Palliative Care, National Cancer Centre Singapore, Singapore, Singapore.

**Keywords:** biliary colic, cognitive impairment, end of life care, palliative care, pancreatic cancer

## Abstract

**Introduction:**

Gallbladder distension secondary to malignant biliary obstruction is an under-recognized cause of abdominal pain in advanced pancreatic cancer and may present atypically outside the right upper quadrant. Pain assessment can be further complicated in older adults with cognitive impairment and superimposed delirium.

**Case Presentation:**

We present an 83-year-old female with locally advanced pancreatic adenocarcinoma and baseline cognitive impairment, admitted to an acute tertiary hospital with painless jaundice. She subsequently developed persistent right iliac fossa pain that was poorly responsive to escalating opioid therapy despite empirical treatment for urinary tract infection and constipation. The inpatient palliative care team was consulted to assist with symptom control. Delirium on a background of cognitive impairment complicated pain assessment. Imaging performed to clarify the cause of escalating pain revealed a grossly dilated gallbladder extending into the mid abdomen, likely due to malignant biliary obstruction. Following multidisciplinary discussions and conversations with family regarding goals of care, invasive interventions were deferred. Pain control improved with opioid titration and the addition of antispasmodic therapy. The patient later developed sepsis and died during the admission.

**Discussion:**

This case highlights key learning points for palliative care practice. Atypical and opioid-refractory pain should prompt reconsideration of pain etiology rather than reflexive opioid escalation. Cognitive impairment and delirium necessitate iterative, multimodal assessment, while caregiver concerns regarding opioid use require proactive engagement. Opioids alone may be insufficient for colicky biliary pain, underscoring the importance of mechanism-based adjuvant strategies. When aligned with goals of care, judicious use of imaging at the end of life can clarify pain etiology and guide proportionate, comfort-focused management.

**Conclusion:**

In advanced cancer, particularly in patients with cognitive impairment and delirium, careful assessment of pain mechanisms, caregiver engagement, and proportionate investigations are essential to optimize comfort and support individualized end-of-life care.

## Introduction

Advanced pancreatic cancer is frequently complicated by pain and biliary obstruction. While abdominal pain is common, pain arising from gallbladder distension secondary to malignant obstruction is rarely emphasized in the palliative care literature, particularly when it presents atypically outside the right upper quadrant. Failure to recognize this mechanism may lead to inappropriate opioid escalation with limited symptomatic benefit.^1^

Pain assessment is further complicated in older adults with cognitive impairment, particularly when delirium develops during acute hospitalization. In such settings, unreliable symptom reporting, caregiver concerns about opioid use, and diagnostic uncertainty can contribute to suboptimal symptom control.^2,3^ Although palliative care prioritizes comfort and avoidance of nonbeneficial interventions, targeted investigations may still be appropriate when they directly inform symptom management and support goal-concordant decision-making. However, the role of diagnostic imaging in end-of-life care remains underdiscussed.^4^

This case addresses these gaps by illustrating an atypical presentation of gallbladder-related pain in advanced pancreatic cancer, the challenges of pain assessment in delirium superimposed on cognitive impairment, and how judicious imaging guided mechanism-based symptom management and informed shared-decision-making within an acute hospital palliative care consult model.

## Case Presentation

An 83-year-old female with a background of diabetes, hypertension, hyperlipidemia, and mild cognitive impairment was diagnosed with locally advanced pancreatic adenocarcinoma and opted for best supportive care due to poor performance status. She was homebound, required assistance with activities of daily living, and was under home hospice care.

She presented to an acute tertiary hospital in Singapore with a 1–2 day history of painless jaundice, tea-colored urine, and pale stools. Obstructive jaundice due to her pancreatic cancer was the initial working diagnosis, and she was admitted under the Medical Oncology Service.

The inpatient palliative care team was consulted on day 3 of admission to assist with symptom management. She developed right iliac fossa (RIF) discomfort, with initial differentials including constipation and urinary tract infection. She had acute urinary retention with mixed bacterial growth on urine culture and was empirically treated with antibiotics. Bowels were cleared, and an indwelling catheter was inserted. Pain management was initiated with a subcutaneous (SC) infusion of fentanyl at 5 mcg/hour, with limited response, requiring regular review and dose escalation.

During this period, she developed hyperactive delirium, attributed to metabolic derangements and pain on a background of cognitive impairment. Computed tomography (CT) scan of the brain excluded acute intracranial causes, and sublingual olanzapine was prescribed as needed for agitation. Pain assessment relied on clinical observation, caregiver input, and the Pain Assessment in Advanced Dementia scale.

By day 9, despite fentanyl escalation to 25 mcg/hour, her pain remained poorly controlled. It was described as episodic and colicky, now with voluntary guarding on examination. Given escalating opioid requirements and unclear pain etiology, discussions were held with the oncology team and family regarding further imaging to guide symptom-focused care.

A CT scan of the abdomen and pelvis revealed a grossly dilated gallbladder extending into the mid-abdomen superior to the iliac crest due to malignant obstruction of the biliary system. No evidence of appendicitis or abscess was seen, and moderate ascites was noted. A CT scan performed one year earlier had shown an ill-defined hypodense lesion at the neck of the pancreas with associated main pancreatic duct dilatation and distal parenchymal atrophy but a normal gallbladder. The interval development and anatomical displacement of the gallbladder provided a plausible explanation for patient’s RIF pain. Percutaneous cholecystostomy was considered but deferred after multidisciplinary discussion of her overall condition and goals of care.

She subsequently developed a fever and was treated with intravenous (IV) antibiotics for presumed hepatobiliary sepsis. Fentanyl was titrated to a final dose of 35 mcg/hour, and regular SC Hyoscine Butylbromide was added as an adjuvant for colicky pain with improved comfort. Throughout the admission, regular conversations were held with her next of kin, establishing a Do Not Resuscitate order and ward-based maximal management, including time-limited trials of IV antibiotics. With improved understanding of the pain mechanism, caregivers agreed to comfort-focused management and declined invasive interventions. The patient deteriorated and died peacefully in hospital.

## Discussion

This case highlights several challenges in the care of terminally ill patients:

### Atypical pain presentation and diagnostic ambiguity

While abdominal pain in pancreatic cancer is common, gallbladder-related colic is under-recognized. The two most common mechanisms leading to pain are pancreatic neuropathy resulting from perineal tumor invasion or nerve impingement and pancreatic duct obstruction, which increases intraductal pressure and leads to pancreatic enzyme deficiency, causing malabsorption and postprandial pain.^5^

In this case, the patient presented with RIF pain despite the primary malignancy being pancreatic head cancer. Pain localization and characteristics were suggestive of colic but were eventually traced to a dilated gallbladder on imaging. From a palliative care perspective, this highlights the importance of a broad differential diagnosis when symptom patterns are atypical or poorly responsive to treatment, rather than defaulting to opioid escalation.^6^

### Difficulty in clinical assessment due to cognitive impairment

Many older adults, including those with mild to moderate cognitive impairment, can reliably report pain using standardized pain intensity scales. However, others experience cognitive impairment that hampers their ability to communicate pain. For example, delirium occurs in 14–18% of elderly patients hospitalized for acute illness, and dementia affects as many as 50% of adults aged 85 years and older. These conditions pose significant barriers to pain assessment and put elderly patients at high risk of undertreated pain.^7^

In the above case, obtaining reliable symptom descriptions was limited by the patient’s short-term memory loss and fluctuating delirium, complicating pain assessment and treatment evaluation. The objective pain assessment tool, PAINAD, was used to assess her pain and response to treatment. A systematic review of pain assessment tools for patients with dementia concluded that there is no gold standard. Overall, evidence about their reliability, validity, and clinical utility remains limited, and no single tool can be universally recommended.^8^

This underscores a core palliative care principle of repeated, multimodal assessment, combining observational tools, clinical examination, and caregiver input to guide symptom management when self-report is unreliable.^9^

### Opioid hesitancy from caregivers

Patients’ family members may, at times, function as a barrier by voicing concerns about the patient taking an opioid. Caregivers’ reluctance to administer opioids can affect the adequacy of cancer pain control. Their perceptions about opioid dependence, tolerance, and side effects, as well as their implications for disease progression, may further influence patients’ use of opioid analgesics.^10^ In this case, family resistance delayed adequate dosing and influenced perception of the patient’s deterioration.

Health care professionals play an essential role in increasing patient and caregiver acceptance of opioids. Targeting misperceptions through discussions with patients and caregivers can lead to better compliance and optimal cancer pain control. Increasing public awareness of opioid analgesics may also help to destigmatize their use and support acceptance of their role in cancer pain management.^11^

Within palliative care practice, ongoing caregiver engagement and education are essential to shared decision-making. Clarifying goals of opioid use as symptom relief rather than life-shortening therapy helps align treatment decisions with patient-centered goals of care. Addressing these concerns early can also prevent misattribution of clinical deterioration to opioid use rather than disease progression.^12^

### Limitations of opioids in biliary colic

Traditional teaching has suggested that morphine increases pressure in the sphincter of Oddi and, therefore, should be avoided in patients with biliary colic.^13^ However, a previous review concluded that the sphincter of Oddi is sensitive to all opioids. Despite this theoretical concern, there are no outcome-based studies showing opioids causing or aggravating biliary colic, and they can therefore still be used in these patients.^14^

Opioids may not adequately control colicky pain caused by biliary obstruction, which may respond to non-steroidal anti-inflammatory drugs (NSAIDs) or antispasmodics. This underscores the need for proper identification of pain etiology before escalation. A Cochrane review supports the finding that NSAIDS are superior to placebo in relieving pain but not superior to opioids nor antispasmodics. However, current available studies are insufficient to establish equivalence in pain relief between these drug classes.^15^

This reflects a palliative care emphasis on mechanism-based analgesia, where adjuvant therapies are introduced according to pain pathophysiology rather than reliance on opioid escalation alone.^16^

### Role of imaging at the end of life

Gishen and Trotman first reported the use of diagnostic imaging in palliative care in 2009, where in-house ultrasound at the bedside in a specialist palliative care unit was used to mark ascites before paracentesis and pleural fluid before chest drain insertion.^17^ Although the overarching goal of end-of-life care is to avoid unnecessary or burdensome interventions, diagnostic imaging may still be required to track disease progression and direct care management.^4^ However, health care workers often associate end-of-life care primarily with supportive and comforting measures and may not recognize the potential benefits and role of medical imaging, instead viewing it as aligned with curative and potentially uncomfortable interventions.^18^

As demonstrated in this case, even in palliative patients, imaging can play a vital role in guiding symptom management, especially when clinical response is suboptimal or diagnosis is unclear. Imaging enabled clarification of pain etiology, informed targeted symptom-focused treatment, and supported decisions to avoid invasive interventions. This aligns with the palliative care principle of proportionality, where investigations are justified when they directly inform comfort-focused management and support goal-concordant decision-making.^19^

## Conclusion

In end-of-life care, particularly for cognitively impaired patients, a high index of suspicion is needed for atypical sources of pain. This case illustrates the importance of targeted diagnostics to optimize comfort, the need to manage caregiver concerns around opioid use, and the limitations of opioids in specific types of pain, such as biliary colic. Applying palliative care principles of mechanism-based symptom management, shared decision-making, and proportionality enabled care to remain focused on comfort while avoiding nonbeneficial interventions.
